# Asymptomatic *Leishmania* infection in HIV-positive outpatients on antiretroviral therapy in Pernambuco, Brazil

**DOI:** 10.1371/journal.pntd.0009067

**Published:** 2021-01-21

**Authors:** Diego Lins Guedes, Alda Maria Justo, Walter Lins Barbosa Júnior, Elis Dionísio da Silva, Samuel Ricarte de Aquino, Manoel Sebastiao da Costa Lima Junior, Ulisses Montarroyos, Gilberto Silva Nunes Bezerra, Amanda Virginia Batista Vieira, Valéria Rêgo Alves Pereira, Zulma Maria de Medeiros

**Affiliations:** 1 Curso de Medicina, Núcleo de Ciências da Vida, Centro Acadêmico do Agreste, Universidade Federal de Pernambuco, Caruaru, Brasil; 2 Departamento de Parasitologia, Instituto Aggeu Magalhães, Fundação Oswaldo Cruz, Recife, Brasil; 3 Núcleo de Pós-Graduação, Faculdade de Ciências Médicas, Universidade de Pernambuco, Recife, Brasil; 4 Departamento de Imunologia, Instituto Aggeu Magalhães, Fundação Oswaldo Cruz, Recife, Brasil; 5 Hospital Universitário, Universidade Federal do Vale do São Francisco, Petrolina, Brasil; Addis Ababa University, ETHIOPIA

## Abstract

**Background:**

Visceral leishmaniasis (VL) in HIV-positive individuals is a global health problem. HIV-*Leishmania* coinfection worsens prognosis and mortality risk, and HIV-*Leishmania* coinfected individuals are more susceptible to VL relapses. Early initiation of antiretroviral therapy can protect against *Leishmania* infection in individuals living in VL-endemic areas, and regular use of antiretrovirals might prevent VL relapses in these individuals. We conducted a cross-sectional study in Petrolina, Brazil, an VL-endemic area, to estimate the prevalence of asymptomatic *Leishmania* cases among HIV-positive outpatients.

**Methods:**

We invited any HIV-positive patients, aged ≥ 18-years-old, under antiretroviral therapy, and who were asymptomatic for VL. Patients were tested for *Leishmania* with enzyme-linked immunosorbent assays (ELISA)-rK39, immunochromatographic test (ICT)-rK39, direct agglutination test (DAT), latex agglutination test (KAtex), and conventional polymerase chain reaction (PCR). HIV-*Leishmania* coinfection was diagnosed when at least one VL test was positive.

**Results:**

A total of 483 patients were included. The sample was predominantly composed of single, < 48-years-old, black/*pardo*, heterosexual males, with fewer than 8 years of schooling. The prevalence of asymptomatic HIV-*Leishmania* coinfection was 9.11% (44/483). HIV mono-infected and HIV-*Leishmania* coinfected groups differed statistically significantly in terms of race (p = 0.045), marital status (p = 0.030), and HIV viral load (p = 0.046). Black/*pardo* patients, married patients, and those with an HIV viral load up to 100,000 copies/ml presented higher odds for HIV-*Leishmania* coinfection.

**Conclusions:**

A considerable number of asymptomatic *Leishmania* cases were observed among HIV-positive individuals in a VL-endemic area. Given the potential impact on transmission and health costs, as well as the impact on these coinfected individuals, studies of asymptomatic *Leishmania* carriers can be useful for guiding public health policies in VL-endemic areas aiming to control and eliminate the disease.

## Author summary

Every year up to 90,000 new cases of visceral leishmaniasis (VL) occurs globally. One of the most neglected diseases, VL is endemic in 60 countries in four continents. Brazil is one of seven countries in which 90% of all cases in the world occur. When co-occurring with HIV, VL tends to be more severe than in HIV-negative persons, leading to worse prognosis and frequent relapses. In this study HIV-positive outpatients, aged 18 years or older, under antiretroviral therapy, who were asymptomatic for VL, were tested for *Leishmania*. We observed a prevalence of 9.11% (44/483) of HIV-*Leishmania* coinfection. For those HIV-positive/*Leishmania*-negative individuals who live in VL-endemic areas, early initiation of antiretroviral therapy can be a protective factor against *Leishmania* infection. On the other hand, for HIV-*Leishmania* coinfected individuals who regularly use antiretrovirals might prevent VL relapses. Studies centering on asymptomatic *Leishmania* carriers can be useful for guiding public health measures in VL-endemic areas seeking to control and eliminate the disease.

## Introduction

One of the most neglected diseases [1], visceral leishmaniasis (VL) is a parasitic disease that mostly affects tropical and subtropical regions [2,3]. Endemic to more than 60 countries, it is estimated that 50,000–90,000 cases occur annually worldwide [4]. Brazil and six other countries account for about 90% of all cases [3]. In the American continent, the majority of VL-positive individuals reside in Brazil [5]. Most of VL cases in Brazil are notified in the Northeast region, and Pernambuco is a VL-endemic area [6].

VL is considered to be an opportunistic infection for those living with the human immunodeficiency virus (HIV) [7]. HIV-positive individuals who live in VL-endemic areas have an increased risk of *Leishmania* infection as compared to HIV-negative individuals [8]. When VL occurs in HIV-infected individuals, the prognosis is typically poor and the mortality rate is high [9]. Consequently, several countries have performed studies to estimate the prevalence of *Leishmania* infection in HIV-positive individuals [10–15].

Asymptomatic *Leishmania* infected individuals, despite the typically low parasite load, might contribute to maintaining the transmission cycle of *Leishmania* parasites in endemic regions during episodes of increased parasite load and disease relapse [16,17]. In Brazil, due to the high prevalence of HIV-*Leishmania* coinfection observed in previous studies [10,15,18], and due to the other potential implications (e.g. frequent relapses, mother-to-child transmission [19]), testing for *Leishmania* should be strongly recommended for all HIV-positive individuals. In addition, once HIV-*Leishmania* coinfection is diagnosed, early start of highly active antiretroviral therapy (HAART) should be recommended as a protective factor against VL relapses [20]. Moreover, for those HIV-positive individuals who are negative for leishmaniasis and are living in VL-endemic areas, HAART could decrease the risk of *Leishmania* infection [21].

The Brazilian national HIV/AIDS program assists all persons who live with HIV with HAART, free of charge, and in the same way it provides treatment for all individuals affected by VL. Despite the impact on the public health system, there have been few studies following up these HIV-*Leishmania* coinfection cases. Most of these studies have focused on hospitalized patients. However, testing for *Leishmania* in HIV-positive outpatients in VL-endemic areas may be useful for guiding health policies aiming to control and eliminate leishmaniasis, and it could improve treatment and outcomes for those affected by the disease.

In this study, we aimed to estimate the prevalence of asymptomatic *Leishmania* cases in HIV-positive outpatients under continuous use of HAART, in a VL-endemic area, Pernambuco, in Northeast Brazil.

## Methods

### Ethics statement

The study was approved by the research ethics committee of Instituto Aggeu Magalhães, Fiocruz Pernambuco (approval number 51235815.0.0000.5190). All subjects were adults and provided written, informed consent. This study was conducted in accordance with the Declaration of Helsinki.

### Study design and sample

We performed a cross-sectional study aiming to estimate the prevalence of HIV-*Leishmania* coinfection in HIV-positive outpatients from the municipality of Petrolina, in the state of Pernambuco, Brazil. Petrolina is an VL-endemic area from where most VL cases in the state of Pernambuco are reported [22]. The study was conducted in a public HIV outpatient clinic, which serviced about 600 individuals regularly using HAART at the time of this investigation.

The study population included any HIV-positive patients, using HAART, aged 18-years-old or older. Individuals under treatment for VL and those with current VL symptoms were excluded. Based on a previous prevalence study in Pernambuco [18], the minimum sample size was calculated to be 159 individuals for a 95% confidence interval (Epi Info 7.2.3.0 software, https://www.cdc.gov/epiinfo/index.html). We publicly invited patients at this outpatient clinic to participate in the study. Many of them demonstrated interest in knowing their serological status for *Leishmania*, as they resided in an endemic area. Due to the high demand, we decided to include anyone who requested to be tested. Therefore, there was a higher number of participants recruited compared to the originally calculated sample size. Each participant received the results of the tests done in the study.

### Data collection and laboratory procedures

After the interview and physical examination, peripheral venous blood and urine samples were collected from the patients at the same time when the samples were taken for analyzing the lymphocyte T CD4+ (LTCD4+) count or HIV viral load. The samples were stored, processed, and analyzed at Fiocruz Pernambuco, a referral public research center. Participants were tested for VL with enzyme-linked immunosorbent assays (ELISA)-rK39, immunochromatographic test (ICT)-rK39, direct agglutination test (DAT), latex agglutination test (KAtex) and polymerase chain reaction (PCR) test. All HIV-positive individuals with at least one positive test for VL were considered HIV-*Leishmania* coinfection cases. Hemogram, biochemistry, LTCD4+ count, and HIV viral load data were obtained from the medical records.

For the ELISA-rK39 assays, the commercial recombinant rK39 antigen was purchased from Rekom Biotech (Granada, Spain) and the assays were essentially carried out as previously described by Scalone et al [23] and Abass et al [24]. For ICT-rK39, we used the OnSite Leishmania IgM/IgG Combo test (CTK Biotech, Inc., Poway, CA, USA) following the manufacturer’s instructions.

For DAT, we used a freeze-dried antigen from Biomedical Research (Amsterdam, The Netherlands) and titers of 1:3,200 or higher were considered to indicate a positive test [25]. *Leishmania* antigen was detected in urine by means of the KAtex kit (Kalon Biological Ltd., Guildford, UK) according to the manufacturer’s instructions.

For PCR, we targeted the kinetoplast DNA of *Leishmania* (kDNA). We used the following primers: 150 5’-GGG(G/T)AGGGGCGTTCT(C/G)CGAA3’ and 152 5’-(C/G)(C/G)(C/G)(A/T)CTAT(A/T) TTACACCAACCCC-3’, which amplify a fragment of 120 bp for all *Leishmania* species. Details on the PCR conditions were described by Souza et al [26]. To confirm the species (*L*. *Infantum*), we used primers RLC2 5’-GGGAAATTGGCCTCCCTGAG-3’ and FLC2 5’-GTCAGTGTCGGAAACTAATCCGC-3’, which amplify a product of 230 bp, according to Gualda et al [27]. The results were analyzed by electrophoresis in 1.5% agarose gels stained with ethidium bromide and were visualized under ultra-violet light.

### Statistical analysis

Data were entered and stored on spreadsheets using Microsoft Excel Professional Plus 2016 software (Microsoft Corp., Redmond, WA, USA). Data analysis was performed in Stata SE 12.0 software for Windows (StataCorp, College Station, TX, USA).

Frequencies and means with 95% confidence interval of the variables of interest were obtained. We compared an HIV mono-infected and an HIV-*Leishmania* coinfected group. For binary/categorical variables, the chi-square test was used (significance level p < 0.05). For continuous variables, we used the two-sample Wilcoxon rank-sum (Mann-Whitney) test. Variables that yielded a p-value ≤ 0.1 in univariate analysis were included in a multivariate analysis using logistic regression.

### Results

Of 487 HIV-positive individuals who agreed to enroll in the study, three individuals were excluded as they presented typical symptoms of VL and one individual was excluded for not using HAART. The study population was composed mainly of single (45.5%), black/*pardo* (83.4%), heterosexual (58.2%) men (61.3%) with 8 or fewer years of schooling (48.24%). About 3% reported using intravenous drugs (Table 1).

**Table 1:** Comparison of epidemiological characteristics between HIV-*Leishmania* coinfected and HIV mono-infected groups (univariate analysis) among HIV-positive individuals tested for *Leishmania* in Petrolina, Brazil.

Variables	HIV mono (N = 439)		HIV-*Leishmania* (N = 44)		p-value
	n	(%)	n	(%)	
**Gender**					
Female	170	(38.7)	17	(38.6)	
Male	269	(61.3)	27	(61.4)	0.991
**Age (years)**					
18–27	70	(15.9)	7	(15.9)	
28–37	109	(24.8)	7	(15.9)	
38–47	140	(31.9)	17	(38.6)	0.582
48 or more	120	(27.3)	13	(29.5)	
**Years of schooling**					
0–8	211	(48.1)	22	(50.0)	0.751
9–11	162	(36.9)	14	(31.8)	
12 or more	66	(15.0)	8	(18.2)	
**Marital status**					
Separated/divorced/widowed	65	(14.8)	2	(4.5)	
Married/stable union	168	(38.3)	28	(63.6)	0.003
Single	206	(46.9)	14	(31.8)	
**Race**					
White	73	(16.6)	1	(2.3)	
Black/*pardo*	336	(83.4)	43	(97.7)	0.039
Indigenous	1	(0.2)	0	(–)	
**Sexual orientation**					
Did not inform	86	(19.6)	7	(15.9)	
Heterosexual	257	(58.5)	24	(54.5)	0.576
Homosexual	76	(17.3)	9	(20.4)	
Bisexual	20	(4.6)	4	(9.1)	
**Use of intravenous drugs**					
No	426	(97.0)	43	(97.7)	0.795
Yes	13	(3.0)	1	(2.3)	
**Dogs at home**					
No	274	(91.3)	165	(90.2)	0.665
Yes	26	(8.7)	18	(9.8)	

All percentages are column percentages

*Pardo* is a specific Brazilian self-declared race, non-white

The prevalence of HIV-*Leishmania* coinfection was 9.11% (44/483). The highest positivity by VL test was seen with DAT (3.53%), followed by ELISA-rK39 (2.48%), and PCR kDNA (2.28%) (Table 2). Two individuals tested positive with DAT and KAtex, and one person tested positive with DAT and rK39. In addition, of the individuals who tested positive for *Leishmania* spp, three (6.8%) reported having had previous diagnoses of VL, and all of whom reported having been treated.

**Table 2:** Prevalence of asymptomatic HIV-*Leishmania* coinfection cases in outpatients from Petrolina, Brazil, according to VL test done.

VL tests	Positivity	Prevalence (%)
ELISA-rK39	12/483	2.48
rK39-ICT	5/470	1.06
DAT	17/482	3.53
KAtex	2/483	0.41
PCR kDNA	11/482	2.28
DAT and KAtex	2/482	0.41
DAT and rK39-ICT	1/470	0.21
**Total (at least one positive test)**	**44/483**	**9.11**

Regarding general laboratory findings, all results were compatible with the reference standards (Table 3). When the two groups were compared, we observed statistically significant differences in terms of marital status (p = 0.003) and race (p = 0.039), with HIV-*Leishmania* coinfection being more frequent in married and black/*pardo* individuals. No general laboratory characteristic showed a statistically significant difference between the groups. In terms of HIV infection status (Table 4), the LTCD4+ count was greater than 350 cells/mm^3^ in 74.4% of the general sample, with no difference between the two groups. Most individuals (73.4%) had un undetectable HIV viral load. When we correlated the LTCD4+ count with the VL tests performed, we observed no statistically significant differences.

**Table 3:** Comparison of general laboratory characteristics between HIV-*Leishmania* coinfected and HIV mono-infected groups (univariate analysis) among HIV-positive individuals tested for *Leishmania* in Petrolina, Brazil.

Variables	HIV mono		HIV-*Leishmania*		p-value
	Mean	(95CI)	Mean	(95CI)	
White blood cells (cells/mm^3^)	5793	(5583–6002)	5325	(4770–5881)	0.289
Hemoglobin (g/dL)	13.8	(13.6–14.0)	13.7	(13.0–14.4)	0.706
Platelets (x10^3^) (cells/mm^3^)	255	(247–264)	263	(238–288)	0.586
AST (U/L)	31.3	(29.1–33.5)	31.1	(26.4–36.0)	0.880
ALT (U/L)	29.6	(27.7–31.5)	26.8	(22.1–31.4)	0.918
Creatinine (mg/dL)	0.82	(0.80–0.85)	0.90	(0.82–0.97)	0.188

95CI, 95% confidence interval; AST, aspartate aminotransferase; ALT, alanine aminotransferase

**Table 4:** Comparison of HIV infection-related characteristics between HIV-*Leishmania* coinfected and HIV mono-infected groups (univariate analysis) among HIV-positive individuals tested for *Leishmania* in Petrolina, Brazil.

Variables	HIV mono		HIV-*Leishmania*		p-value
	n	(%)	n	(%)	
**HIV viral load (copies/ml)**					
Undetectable (<50)	326	(74.4)	28	(63.6)	0.099
Up to 100,000	90	(20.5)	15	(34.1)	
100,000 or more	22	(5.0)	1	(2.3)	
**LTCD4+ count (cells/mm**^**3**^**)**					
Up to 200	43	(10.1)	5	(11.9)	
200 to 350	66	(15.5)	6	(14.3)	
350 or more	318	(74.5)	31	(73.8)	0.922

All percentages are column percentages; LTCD4+, lymphocyte T CD4+

Marital status, race, and HIV viral load remained significant in the multivariate model (Table 5). Black/*pardo* individuals were at an increased risk (odds ratio, OR: 7.85; p = 0.044) of being HIV-*Leishmania* co-infected, as compared with white individuals. Marriage/stable unions and a detectable HIV viral load up to 100,000 copies/mL were also associated with an increased risk (OR: 5.12, p = 0.029 and OR: 2.01, p = 0.047, respectively).

**Table 5:** Odds ratios for asymptomatic HIV-*Leishmania* coinfection based on multivariate analysis.

Variable	OR	95CI	p-value
**Race**			
White	1		
Black/*pardo*	7.85	1.05–58.39	**0.044**
**Marital status**			
Separated/Divorced/Widow	1		
Married/Stable union	5.12	1.17–22.29	**0.029**
Single	1.99	0.44–9.09	0.374
**HIV viral load (copies/ml)**			
Undetectable (<50)	1		
Up to 100,000	2.01	1.01–4.05	**0.047**
100,000 or more	0.70	0.09–5.60	0.740

OR odds ratio; 95CI 95% confidence interval

## Discussion

This study focused on HIV-*Leishmania* coinfection in outpatients in Pernambuco, which had not been reported previously. The prevalence of asymptomatic HIV-*Leishmania* coinfection was 9.11% (44/483). There were statistically significant differences between the HIV mono-infected and HIV-*Leishmania* coinfected groups in term of race (p = 0.045), marital status (p = 0.030), and HIV viral load (p = 0.046). Black/*pardo* patients, married patients, and those with an HIV viral load up to 100,000 copies/mL presented higher odds for HIV-*Leishmania* coinfection.

The prevalence of asymptomatic HIV-*Leishmania* coinfection in this study (9.1%) was lower than that in a previous study in Pernambuco of hospitalized HIV-positive patients who were tested for VL (16.9%) [18]. This previous study involved three referral hospitals for infectious diseases that serviced the entire state. Despite the higher percentage, compared with the current study, there were fewer cases (35 vs 44) and we tested more individuals (483 vs 207). A similar study in Minas Gerais, Southeastern Brazil, observed a prevalence of asymptomatic *Leishmania* infection in HIV-positive individuals of 20% [15]. Minas Gerais is a Brazilian state with high VL endemicity, and it would be expected to have a higher prevalence than that observed in our study. In the Metema district of Northwestern Ethiopia, a pilot study in HIV-infected adults identified a prevalence of 12.8% in males and 4.2% in females for asymptomatic HIV-*Leishmania* coinfection cases, based on the same tests used in the present study [28].

In the VL tests used, we observed a low coincidence of results. Based on previous studies [15,28,29], a large variation in the test results could be expected. Furthermore, the sample consisted of HIV-positive individuals with no suspicion of VL. The highest prevalence rate was observed with the DAT, a test with high sensitivity that is considered to be a good diagnostic tool for immunocompromised individuals [30,31]. The DAT has also showed the highest prevalence rate in a previous prevalence study in Pernambuco with symptomatic patients [18]. We observed a low prevalence with the ICT-rK39 rapid test (1%). Apparently, this is not the best serological screening test for individuals living in *L*. *infantum*-endemic regions [15,18,29,30]. Despite the reduced sensitivity of serological tests for VL in HIV-positive individuals [8,30], this type of test should not be excluded, as a positive result should be considered when associated with clinical features [32]. Serological tests are important as screening tests for VL, particularly in VL-endemic areas and for detection of asymptomatic HIV-*Leishmania* coinfected persons, as they normally have higher LTCD4+ counts. In our study, most participants had an LTCD4+ count exceeding 350 cells/mm^3^, and thus they had potentially similar humoral response as HIV-negative persons.

In terms of epidemiological aspects, we observed statistically significant differences for race and marital status. Indeed, in Brazil race and poverty are strongly connected, and most people affected by neglected tropical diseases live in low income regions [33], such as the region in which this study was performed.

The mean LTCD4+ count in HIV-*Leishmania* co-infected individuals was similar to that observed in the HIV mono-infected group. In the present study, almost three-quarters of all individuals presented with LTCD4+ exceeding 350 cells/ mm^3^. Most participants from the previous study of hospitalized patients in Pernambuco had LTCD4+ counts lower than 200 cells/mm^3^ in both groups (VL-HIV and HIV) [18]. New cohort studies with paired samples may better explain the behavior of *Leishmania* infection in our population. In the present study, all HIV-positive individuals were on HAART, which explains the higher LTCD4+ count, and which might be a protective factor against developing VL.

We observed that, a detectable viral load, although lower than 100,000 copies/ml, was associated with *Leishmania* infection. As all patients were asymptomatic, this could indicate an initial HAART failure or irregular use of the treatment. In the previous study of hospitalized VL-HIV coinfected patients, only 16% had an undetectable viral load [18], while in the present study the viral load was undetectable in 63% of the coinfected individuals. Since regular use of HAART usually increases the LTCD4+ count, which, in turn, is a protective factor against VL relapses, campaigns to encourage the regular use of antiretrovirals should be intensified among individuals living with HIV in VL-endemic areas. In Brazil, to date, only secondary prophylaxis for VL is recommended, and the only one marker used to guide this prophylaxis is the LTCD4+ count.

The factors determining maintenance of an asymptomatic VL state have not yet been established. This balance between the parasite infection and the host's immune response, such as, for example, in blood donors or in HIV-AIDS patients, probably extends beyond nutritional status and genetic factors. Due to the increased risk of relapses and the poor prognosis, it is important for HIV-positive persons, particularly those living in VL-endemic areas, to know about a previous *Leishmania* infections. Identification of new markers or tests that might delimit active disease, suggest cure, and predict relapses is urgent. It may be challenging to distinguish active VL cases from another opportunistic infection, and new and less-invasive markers for VL could help health professionals in making more accurate diagnoses and consequently avoiding unnecessary treatments.

## Conclusions

Visceral leishmaniasis remains an important problem in Brazil, particularly in the Northeastern region. We observed a considerable number of asymptomatic *Leishmania* cases in HIV-positive individuals. Studies focusing on health care of asymptomatic individuals could be useful for public health policies in VL-endemic areas, facilitating monitoring of the progress of leishmaniasis control. In addition, at the individual level, it is important to follow up all these HIV-*Leishmania* coinfected persons in terms of VL prophylaxis and treatment, and to predict relapses. Consequently, we strongly recommend testing for *Leishmania* in all HIV-positive individuals in VL-endemic areas.

## References

[1] Okwor I, Uzonna J. Social and Economic Burden of Human Leishmaniasis. Am J Trop Med Hyg. 2016;94: 489–493. 10.4269/ajtmh.15-0408 26787156 PMC4775878

[2] Alvar J, Vélez ID, Bern C, Herrero M, Desjeux P, Cano J, et al Leishmaniasis Worldwide and Global Estimates of Its Incidence. Kirk M, editor. PLoS One. 2012;7: e35671 10.1371/journal.pone.0035671 22693548 PMC3365071

[3] WHO. WHO website—Leishmaniasis: fact sheets. 2019. Available: https://www.who.int/en/news-room/fact-sheets/detail/leishmaniasis

[4] Burza S, Croft SL, Boelaert M, Organização Mundial da Saúde. Leishmaniasis. Lancet. 2018;392: 951–970. 10.1016/S0140-6736(18)31204-2 30126638

[5] Pan American Health Organization. Leishmaniasis: Epidemiological Report of the Americas. 2019 p. 8.

[6] Bezerra JMT, de Araújo VEM, Barbosa DS, Martins-Melo FR, Werneck GL, Carneiro M. Burden of leishmaniasis in Brazil and federated units, 1990–2016: Findings from Global Burden of Disease Study 2016. van Griensven J, editor. PLoS Negl Trop Dis. 2018;12: e0006697 10.1371/journal.pntd.0006697 30188898 PMC6126835

[7] Pasquau F, Ena J, Sanchez R, Cuadrado JM, Amador C, Flores J, et al Leishmaniasis as an opportunistic infection in HIV-infected patients: determinants of relapse and mortality in a collaborative study of 228 episodes in a Mediterreanean region. Eur J Clin Microbiol Infect Dis. 2005;24: 411–418. 10.1007/s10096-005-1342-6 15928908

[8] Alvar J, Aparicio P, Aseffa A, Den Boer M, Canavate C, Dedet J-P, et al The Relationship between Leishmaniasis and AIDS: the Second 10 Years. Clin Microbiol Rev. 2008;21: 334–359. 10.1128/CMR.00061-07 18400800 PMC2292576

[9] Henn GA de L, Ramos Júnior AN, Colares JKB, Mendes LP, Silveira JGC, Lima AAF, et al Is Visceral Leishmaniasis the same in HIV-coinfected adults? Brazilian J Infect Dis. 2018;22: 92–98. 10.1016/j.bjid.2018.03.001 29601790 PMC9428234

[10] Carranza-Tamayo CO, de Assis TSM, Neri ATB, Cupolillo E, Rabello A, Romero GAS. Prevalence of Leishmania infection in adult HIV/AIDS patients treated in a tertiary-level care center in Brasilia, Federal District, Brazil. Trans R Soc Trop Med Hyg. 2009;103: 743–8. 10.1016/j.trstmh.2009.01.014 19232657

[11] Cota GF, de Sousa MR, de Mendonça ALP, Patrocinio A, Assunção LS, de Faria SR, et al Leishmania-HIV co-infection: clinical presentation and outcomes in an urban area in Brazil. PLoS Negl Trop Dis. 2014;8: e2816 10.1371/journal.pntd.0002816 24743472 PMC3990491

[12] Diro E, Lynen L, Ritmeijer K, Boelaert M, Hailu A, van Griensven J. Visceral Leishmaniasis and HIV Coinfection in East Africa. PLoS Negl Trop Dis. 2014;8: e2869 10.1371/journal.pntd.0002869 24968313 PMC4072530

[13] Echchakery M, Nieto J, Boussaa S, El Fajali N, Ortega S, Souhail K, et al Asymptomatic carriers of Leishmania infantum in patients infected with human immunodeficiency virus (HIV) in Morocco. Parasitol Res. 2018;117: 1237–1244. 10.1007/s00436-018-5805-y 29478175

[14] Monge-Maillo B, Norman FF, Cruz I, Alvar J, López-Vélez R. Visceral Leishmaniasis and HIV Coinfection in the Mediterranean Region. Valenzuela JG, editor. PLoS Negl Trop Dis. 2014;8: e3021 10.1371/journal.pntd.0003021 25144380 PMC4140663

[15] Orsini M, Canela JR, Disch J, Maciel F, Greco D, Toledo A, et al High frequency of asymptomatic Leishmania spp. infection among HIV-infected patients living in endemic areas for visceral leishmaniasis in Brazil. Trans R Soc Trop Med Hyg. 2012;106: 283–8. 10.1016/j.trstmh.2012.01.008 22348817

[16] Das VNR, Siddiqui NA, Verma RB, Topno RK, Singh D, Das S, et al Asymptomatic infection of visceral leishmaniasis in hyperendemic areas of Vaishali district, Bihar, India: A challenge to kala-azar elimination programmes. Trans R Soc Trop Med Hyg. 2011;105: 661–666. 10.1016/j.trstmh.2011.08.005 21945327

[17] Molina R, Jiménez M, García-Martínez J, San Martín JV, Carrillo E, Sánchez C, et al Role of asymptomatic and symptomatic humans as reservoirs of visceral leishmaniasis in a Mediterranean context. Schönian G, editor. PLoS Negl Trop Dis. 2020;14: e0008253 10.1371/journal.pntd.0008253 32324738 PMC7200008

[18] Guedes DL, Medeiros Z, Dionísio da Silva E, Martins de Vasconcelos AV, Santana da Silva M, Lopes da Silva MA, et al Visceral Leishmaniasis in Hospitalized HIV-Infected Patients in Pernambuco, Brazil. Am J Trop Med Hyg. 2018;99: 1541–1546. 10.4269/ajtmh.17-0787 30328408 PMC6283492

[19] Argy N, Lariven S, Rideau A, Lemoine A, Bourgeois Moine A, Allal L, et al Congenital Leishmaniasis in a Newborn Infant Whose Mother was Coinfected With Leishmaniasis and HIV. J Pediatric Infect Dis Soc. 2020;9: 277–280. 10.1093/jpids/piz055 31589299

[20] Abongomera C, Diro E, Vogt F, Tsoumanis A, Mekonnen Z, Admassu H, et al The Risk and Predictors of Visceral Leishmaniasis Relapse in Human Immunodeficiency Virus-Coinfected Patients in Ethiopia: A Retrospective Cohort Study. Clin Infect Dis. 2017;65: 1703–1710. 10.1093/cid/cix607 29020196 PMC5848226

[21] López-Vélez R. The impact of highly active antiretroviral therapy (HAART) on visceral leishmaniasis in Spanish patients who are co-infected with HIV. Ann Trop Med Parasitol. 2003;97: 143–147. 10.1179/000349803225002615 14678641

[22] Diniz LFB, de Souza CDF, do Carmo RF. Epidemiology of human visceral leishmaniasis in the urban centers of the lower-middle São Francisco Valley, Brazilian semiarid region. Rev Soc Bras Med Trop. 2018;51: 461–466. 10.1590/0037-8682-0074-2018 30133628

[23] Scalone A, De Luna R, Oliva G, Baldi L, Satta G, Vesco G, et al Evaluation of the Leishmania recombinant K39 antigen as a diagnostic marker for canine leishmaniasis and validation of a standardized enzyme-linked immunosorbent assay. Vet Parasitol. 2002;104: 275–285. 10.1016/s0304-4017(01)00643-4 11836028

[24] Abass E, Bollig N, Reinhard K, Camara B, Mansour D, Visekruna A, et al rKLO8, a Novel Leishmania donovani—Derived Recombinant Immunodominant Protein for Sensitive Detection of Visceral Leishmaniasis in Sudan. PLoS Negl Trop Dis. 2013;7 10.1371/journal.pntd.0002322 23875052 PMC3715527

[25] El Harith A, Kolk AHJ, Leewenburg J, Muigai R, Huigen E, Jelsma T, et al Improvement of a Direct Agglutination Test for Field Studies of Visceral Leishmaniasis. Journal of clinical microbiology. 1988 pp. 1321–1325. 10.1128/JCM.26.7.1321-1325.1988 3410946 PMC266601

[26] Souza NP, de Almeida A do BPF, de Freitas TPT, da Paz RCR, Dutra V, Nakazato L, et al Leishmania (Leishmania) infantum chagasi em canídeos silvestres mantidos em cativeiro, no Estado de Mato Grosso. Rev Soc Bras Med Trop. 2010;43: 333–335. 10.1590/s0037-86822010000300024 20563507

[27] Gualda KP, Marcussi LM, Neitzke-Abreu HC, Aristides SMA, Lonardoni MVC, Cardoso RF, et al New Primers for Detection of Leishmania infantum Using Polymerase Chain Reaction. Rev Inst Med Trop Sao Paulo. 2015;57: 377–83. 10.1590/S0036-46652015000500002 26603223 PMC4660445

[28] van Griensven J, van Henten S, Mengesha B, Kassa M, Adem E, Endris Seid M, et al Longitudinal evaluation of asymptomatic Leishmania infection in HIV-infected individuals in North-West Ethiopia: A pilot study. PLoS Negl Trop Dis. 2019;13: e0007765 10.1371/journal.pntd.0007765 31593563 PMC6799935

[29] de Gouvêa Viana L, de Assis TSM, Orsini M, da Silva AR, de Souza GF, Caligiorne R, et al Combined diagnostic methods identify a remarkable proportion of asymptomatic Leishmania (Leishmania) chagasi carriers who present modulated cytokine profiles. Trans R Soc Trop Med Hyg. 2008;102: 548–555. 10.1016/j.trstmh.2008.02.007 18367221

[30] Cota GF, de Sousa MR, de Freitas Nogueira BM, Gomes LI, Oliveira E, Assis TSM, et al Comparison of parasitological, serological, and molecular tests for visceral leishmaniasis in HIV-infected patients: a cross-sectional delayed-type study. Am J Trop Med Hyg. 2013;89: 570–7. 10.4269/ajtmh.13-0239 23836568 PMC3771302

[31] van Griensven J, Diro E. Visceral Leishmaniasis: Recent Advances in Diagnostics and Treatment Regimens. Infect Dis Clin North Am. 2019;33: 79–99. 10.1016/j.idc.2018.10.005 30712769

[32] Cota GF, de Sousa MR, Demarqui FN, Rabello A. The Diagnostic Accuracy of Serologic and Molecular Methods for Detecting Visceral Leishmaniasis in HIV Infected Patients: Meta-Analysis. Boelaert M, editor. PLoS Negl Trop Dis. 2012;6: e1665 10.1371/journal.pntd.0001665 22666514 PMC3362615

[33] Lindoso JAL, Lindoso AABP. Neglected tropical diseases in Brazil Revista do Instituto de Medicina Tropical de Sao Paulo. Instituto de Medicina Tropical de Sao Paulo; 2009 pp. 247–253. 10.1590/s0036-46652009000500003 19893976

